# Accurate and robust auto‐segmentation of head and neck organ‐at‐risks based on a novel CNN fine‐tuning workflow

**DOI:** 10.1002/acm2.14248

**Published:** 2023-12-21

**Authors:** Shunyao Luan, Kun Wu, Yuan Wu, Benpeng Zhu, Wei Wei, Xudong Xue

**Affiliations:** ^1^ Department of Radiation Oncology Hubei Cancer Hospital, Tongji Medical College Huazhong University of Science and Technology Wuhan China; ^2^ School of Integrated Circuits Laboratory for Optoelectronics Huazhong University of Science and Technology Wuhan China

**Keywords:** convolutional neural network, fine‐tuning, radiotherapy, segmentation

## Abstract

**Purpose:**

Obvious inconsistencies in auto‐segmentations exist among various AI software. In this study, we have developed a novel convolutional neural network (CNN) fine‐tuning workflow to achieve precise and robust localized segmentation.

**Methods:**

The datasets include Hubei Cancer Hospital dataset, Cetuximab Head and Neck Public Dataset, and Québec Public Dataset. Seven organs‐at‐risks (OARs), including brain stem, left parotid gland, esophagus, left optic nerve, optic chiasm, mandible, and pharyngeal constrictor, were selected. The auto‐segmentation results from four commercial AI software were first compared with the manual delineations. Then a new multi‐scale lightweight residual CNN model with an attention module (named as HN‐Net) was trained and tested on 40 samples and 10 samples from Hubei Cancer Hospital, respectively. To enhance the network's accuracy and generalization ability, the fine‐tuning workflow utilized an uncertainty estimation method for automatic selection of candidate samples of worthiness from Cetuximab Head and Neck Public Dataset for further training. The segmentation performances were evaluated on the Hubei Cancer Hospital dataset and/or the entire Québec Public Dataset.

**Results:**

A maximum difference of 0.13 and 0.7 mm in average Dice value and Hausdorff distance value for the seven OARs were observed by four AI software. The proposed HN‐Net achieved an average Dice value of 0.14 higher than that of the AI software, and it also outperformed other popular CNN models (HN‐Net: 0.79, U‐Net: 0.78, U‐Net++: 0.78, U‐Net‐Multi‐scale: 0.77, AI software: 0.65). Additionally, the HN‐Net fine‐tuning workflow by using the local datasets and external public datasets further improved the automatic segmentation with the average Dice value by 0.02.

**Conclusion:**

The delineations of commercial AI software need to be carefully reviewed, and localized further training is necessary for clinical practice. The proposed fine‐tuning workflow could be feasibly adopted to implement an accurate and robust auto‐segmentation model by using local datasets and external public datasets.

## 1 INTRODUCTION

Head and neck cancer (HNC) ranks as the seventh most prevalent cancer worldwide, and is estimated to cause 50,000 deaths in 2018.^1^ Radiotherapy serves as the primary curative treatment option recommended for this disease. Existing evidence suggests that delineating the key anatomical structures in a single HNC patient typically takes approximately 2.7–3 h, with 0.5–1 h specifically dedicated to delineating organs‐at‐risk (OARs).^2^ Currently, this delineation process is primarily performed manually using a treatment planning system (TPS). However, it is important to note that manual delineation is prone to inter‐observer variability, a phenomenon that is closely related to the knowledge, experience, and preferences of the radiation oncologists involved.^3^

Conventional techniques, such as atlas‐based methods^4^, ^5^, ^6^ and hybrid model‐based methods,^7^, ^8^ have been used in clinical practice to improve both the effectiveness and accuracy of the segmentation process. In the atlas‐based approach, segmentation is achieved by aligning a predefined set of manually labeled reference examples with the new images. Meanwhile, hybrid model‐based methods involve statistical analysis of ground truth contours, accompanied by the imposition of prior shape constraints during the segmentation procedure. It is important to recognize that these approaches may face limitations due to the substantial anatomical variations observed in human organs or the inherent local uncertainties associated with deformable registration.^2^

Currently, deep learning, exemplified by deep convolutional neural networks (CNNs), has demonstrated remarkable success in the fields of computer science and medical image analysis. Numerous investigations have harnessed the power of CNNs to perform segmentation tasks on various organs and substructures in the context of radiotherapy across different disease sites and imaging modalities.^9^, ^10^, ^11^, ^12^, ^13^, ^14^, ^15^, ^16^ However, given the intrinsic variability in the size of organs at risk (OARs), especially in cases where specific OARs occupy only a few image slices, it is imperative to evaluate the effectiveness of deep neural network models for OAR segmentation within the head and neck (HN) region. Ibragimov first applied convolutional neural networks to segment OARs in head and neck CT images in radiotherapy. Their results showed a Dice similarity coefficient (DSC) ranging from 37.4% for the chiasm to 89.5% for the mandible.^17^ Another notable effort by Sun et al. involved the development of a two‐step approach, starting with precise localization followed by segmentation, for CT image segmentation involving the eyes and adjacent anatomical structures. This approach demonstrated high accuracy, efficiency, and suitability for clinical use.^18^ Furthermore, Zhu et al. introduced a groundbreaking end‐to‐end, atlas‐free, and fully automated deep learning model for anatomical segmentation in head and neck CT images. This innovative model incorporated a novel coding scheme, 3D squeeze and excitation residual blocks, and a combined loss function. Experimental results showed an average increase of 3.3% in the Dice similarity coefficient (DSC).^19^

As CNNs have been a state‐of‐the‐art segmentation method in radiotherapy, more and more commercial artificial intelligence (AI) software are being introduced into clinical practice. However, a complete subjective and comprehensive evaluation of their performance is lacking. Due to the significant inconsistency between different gold standard training datasets, the automatic segmentations provided by different vendors vary widely. Different network architectures will also lead to discrepancies in prediction results. This will increase the segmentation uncertainty, and the impact on clinical practice cannot be neglected.

In this study, we developed a novel fine‐tuning training workflow based on multi‐scale lightweight residual convolutional neural network with attention module for precise and robust localized segmentation. The contributions of this paper are as follows:

1. The automatic delineations performed by the four commercial AI software were compared with manual contours on the same local HN datasets.
2. We proposed a new multi‐scale lightweight residual convolutional neural network with attention module, which is widely applicable to segment different sizes of OARs.
3. We developed a novel fine‐tuning workflow that uses the uncertainty estimation method to select valuable and diverse samples from the public dataset. This will improve the accuracy and robustness of the local training model.

## 2 MATERIALS AND METHODS

### 2.1 Patient data

Our datasets consist of Hubei Cancer Hospital dataset (50 nasopharyngeal carcinoma samples), Cetuximab Head and Neck Public Dataset (30 samples), and Québec Public Dataset (58 samples). Patients at the Huber Cancer Hospital were immobilized in the supine position with a thermoplastic mask and then underwent a contrast‐enhanced simulated CT scan on the Philips Brilliance Big Bore CT system. The size and thickness of the CT images were 512×512 and 3 mm, and the pixel spacing ranged from 0.7 to 1.1 mm. The number of slices per CT scan varied from 90 to 130 slices.

### 2.2 Segmentation comparisons

The auto‐segmentation results of four commercial AI software were first compared with the manual delineations using 50 samples from local hospitals. These AI software were Manteia (Xiamen, China), PVmed (Guangzhou, China), Haichuang Medical Company (Hangzhou, China), and Linking Med (Beijing, China), respectively. Seven OARs were selected and delineated by two independent radiation oncologists according to the guideline^20^ as gold standards on the CT images using Eclipse (Varian Medical Systems, Palo Alto, CA) treatment planning system, including the brain stem, left parotid gland, esophagus, left optic nerve, optic chiasm, mandible, and pharyngeal constrictor. The original CT images were transferred to the four AI workstations. The segmentation contours were then collected and output to our in‐house written software to test its segmentation performance.

The detailed deep learning methods based on Manteia software was a residue‐UNet with Dice loss, and both encoder and decoder are composed of 5 cascades of residual blocks.^21^ The network of Haichuang software was a deep dilated convolutional neural network, similar to the VGG‐16 model.^22^ The proposed network model of PVmed was based on 3D‐UNet architecture.^23^ To tackle with the segmentation task, Linking Med developed a two‐step segmentation model: a slice classification model was used to classify CT slices into six categories in the craniocaudal direction^24^; then the target categories for different OARs were pushed to the different 3D encoder–decoder segmentation networks, respectively.

### 2.3 Image pre‐processing

In order to train the CNN model by ourselves, image pre‐processing is necessary. Bilinear interpolation was applied to adjust the different pixel spacing to 1 mm. In this way, the slice sizes are smaller or larger than 512×512 pixels. We use a zero‐padding method to uniformly fill the pixel size of all slices to 512×512, or a cropping method to keep the same dimension.

Furthermore, despite the large number of critical organs in the head and neck, in this study we divided the organs of the head and neck into two main categories: soft tissue organs (such as the brainstem, optic chiasm, and optic nerves) and skeletal structures (such as the mandible). Each category underwent specific window widths and window levels. Typically, window widths and levels were set at [−140, 210] Hounsfield Units (HU) for soft tissues and [−350, 1250] HU for skeletal structures. This strategy helps to more robustly capture the diversity and complexity of structures in the head and neck region. Note that we have created dedicated models for each organ‐at‐risk, sharing a common architecture but with different parameters. In practical clinical applications, achieving segmentation for different organs‐at‐risk requires only the input of pre‐trained parameters into the model.

### 2.4 Workflow

**FIGURE 1:**
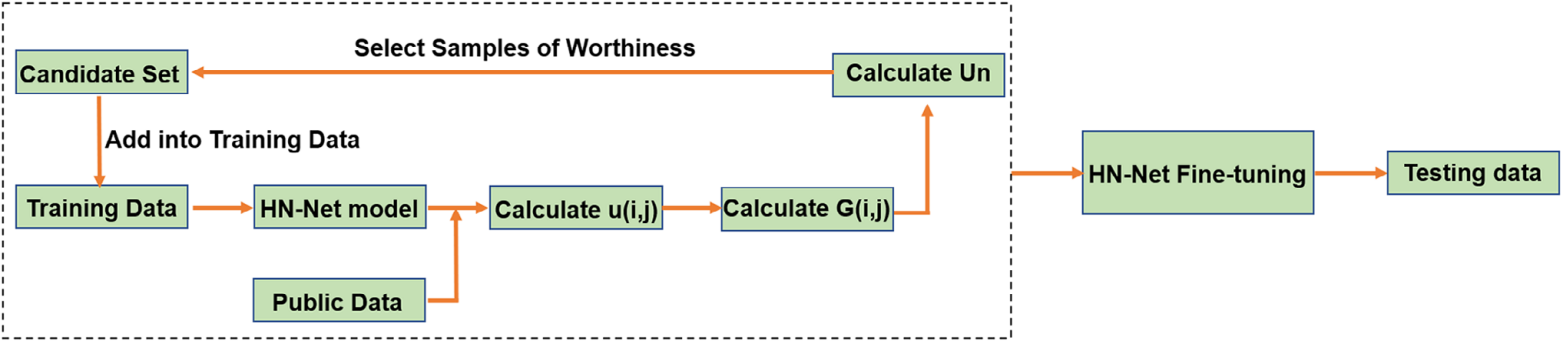
The workflow of establishing a fine‐tuning HN‐Net model.

The novel fine‐tuning training workflow for establishing the local CNN model was shown in Figure 1.

1. The 40 samples of Hubei Cancer Hospital dataset, that is, gold standard datasets, were initially used to train the proposed HN‐Net.
2. To address the model's poor generalization and inadequate training data, we implemented the uncertainty estimation method to select valuable and diverse samples from the Cetuximab head and neck public datasets and added them into the training dataset to fine‐tune the model.
3. The 10 samples of Hubei Cancer Hospital and/or the whole Québec Public Dataset were used as the testing dataset to evaluate the segmentation performance. Table 1, 2, 3, 4 shows the results based on local Hubei Cancer Hospital dataset. The results in Table 5 are based on Québec Public Dataset and 10 samples of Hubei Cancer Hospital.

**TABLE 1:** Dice value comparisons of auto‐segmentations from four commercial AI software (A, B, C and D) with the same testing dataset.

	A	B	C	D	Average
Brain stem	**0.84** ± 0.04	0.79 ± 0.04	0.83 ± 0.04	0.81 ± 0.05	0.82 ± 0.04
Esophagus	**0.80** ± 0.06	0.69 ± 0.06	0.58 ± 0.10	0.74 ± 0.10	0.70 ± 0.08
Mandible	0.84 ± 0.03	**0.86** ± 0.03	0.81 ± 0.04	0.75 ± 0.04	0.81 ± 0.04
Parotid	**0.79** ± 0.06	0.74 ± 0.06	0.69 ± 0.10	0.63 ± 0.08	0.71 ± 0.08
Optic chiasm	**0.52** ± 0.18	0.45 ± 0.16	0.26 ± 0.13	0.36 ± 0.14	0.40 ± 0.14
Optic nerve	0.56 ± 0.12	**0.57** ± 0.13	0.38 ± 0.14	0.41 ± 0.15	0.49 ± 0.13
Pharyngeal constrictor	**0.67** ± 0.06	0.62 ± 0.06	**–**	**–**	0.64 ± 0.08
Average	**0.72** ± 0.08	0.67 ± 0.08	0.59 ± 0.09	0.62 ± 0.09	–

The bold value indicates the optimal value among the four commercial AI software.

**TABLE 2:** HD value comparisons of auto‐segmentations from four commercial AI software (A, B, C and D) with the same testing dataset.

	A(mm)	B(mm)	C(mm)	D(mm)	Average(mm)
Brain stem	**2.5** ± 0.4	2.7 ± 0.4	3.0 ± 0.5	2.7 ± 0.4	2.7 ± 0.4
Esophagus	**2.5** ± 0.8	4.2 ± 1.1	4.2 ± 0.9	3.0 ± 0.9	3.5 ± 0.9
Mandible	7.2 ± 3.0	**6.3** ± 2.2	6.7 ± 2.4	8.6 ± 2.6	7.2 ± 2.5
Parotid	**5.4** ± 2.1	6.5 ± 1.9	7.2 ± 2.5	6.3 ± 2.3	6.4 ± 2.2
Optic chiasm	**4.0** ± 2.4	5.1 ± 2.1	5.7 ± 3.5	4.6 ± 2.3	4.9 ± 2.7
Optic nerve	3.5 ± 2.7	3.5 ± 2.5	3.6 ± 1.9	**2.3** ± 1.7	3.2 ± 2.2
Pharyngeal constrictor	5.8 ± 1.1	**4.3** ± 0.8	**–**	**–**	5.1 ± 1.0
Average	4.4 ± 1.8	4.7 ± 1.6	5.1 ± 2.0	4.6 ± 1.8	–

The bold value indicates the optimal value among the four commercial AI software.

**TABLE 3:** Performance comparison with different network structures, evaluated with dice coefficients.

	AI software	U‐Net^21^	U‐Net++^22^	UNet‐multi‐scale^23^	HN‐Net
Brain stem	0.82^**^	0.87	**0.88**	0.87	0.87
Esophagus	0.70^***^	0.82^**^	**0.83**	0.80^***^	0.83
Mandible	0.81^***^	0.90^*^	0.89^***^	0.87^***^	**0.91**
Parotid	0.71^***^	**0.85**	0.84^*^	0.83^**^	**0.85**
Optic chiasm	0.40^***^	0.56^*^	0.56	0.56^*^	**0.57**
Optic nerve	0.49^***^	0.73	0.74	0.72^**^	**0.74**
Pharyngeal constrictor	0.64^***^	0.74	0.73^*^	0.73^*^	**0.74**
Average	0.65^***^	0.78^*^	0.78^*^	0.77^**^	**0.79**

*Note*: The HN‐Net's segmentation results were compared with those of other four models using paired t‐test. Please note that “^***^” to indicate *p* < 0.001, “^**^” to indicate *p* < 0.01 and “^*^” to indicate *p* < 0.05.

The bold value indicates the optimal value among the five methods.

**TABLE 4:** Auto‐segmentation results for HN‐Net, measured with Dice, Jaccard, Precision, Recall and Hausdorff distance values.

	Dice	Jaccard	Precision	Recall	HD (mm)
Brain stem	0.87	0.78	0.99	0.87	2.2
Esophagus	0.83	0.75	0.88	0.84	1.1
Mandible	0.91	0.86	0.94	0.91	1.4
Parotid	0.85	0.75	0.94	0.82	1.5
Optic chiasm	0.57	0.45	0.69	0.54	4.7
Optic nerve	0.74	0.66	0.82	0.78	2.1
Pharyngeal constrictor	0.74	0.59	0.89	0.63	2.1
Average	0.79	0.69	0.88	0.77	2.6

**TABLE 5:** Auto‐segmentation Dice results for U‐Net, HN‐Net and their corresponding fine‐tuning model.

	U‐Net	U‐Net + fine Tuning	HN‐Net	HN‐Net + fine Tuning
Brain stem	0.80^***^	0.84^**^	0.81^***^	**0.85**
Esophagus	0.76^***^	0.78^*^	0.77^**^	**0.79**
Mandible	0.84^***^	0.87^*^	0.86^**^	**0.88**
Parotid	0.81^***^	0.82^*^	0.81^***^	**0.83**
Optic chiasm	0.48^***^	0.48^***^	0.51^*^	**0.52**
Optic nerve	0.67^***^	0.69^***^	0.69^**^	**0.71**
Average	0.73^***^	0.75^**^	0.74^**^	**0.76**

*Note*: The HN‐Net + fine‐tuning's segmentation results were compared with those of U‐Net, U‐Net + fine‐tuning, and HN‐Net model using paired t‐test. Please note that “^***^” to indicate *p* < 0.001, “^**^” to indicate *p* < 0.01 and “^*^” to indicate *p* < 0.05.

The bold value indicates the optimal value.

### 2.5 HN‐Net architecture

**FIGURE 2:**
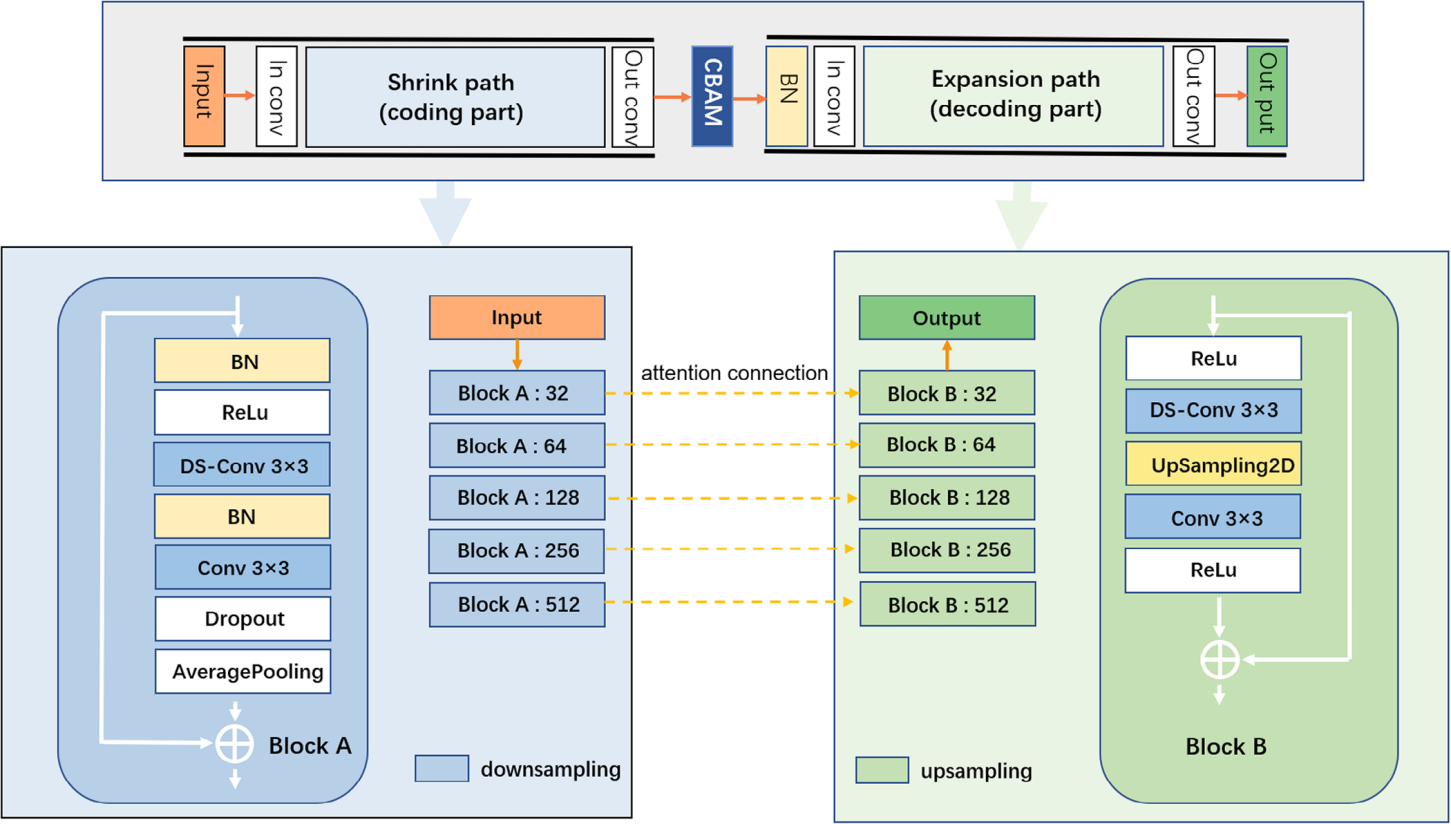
The proposed HN‐Net architecture. BN represents Batch normalization, and DS‐conv represents depthwise separable convolution.

We proposed a multi‐scale lightweight residual convolutional neural network with attention module. A typical encoding‐decoding structure was used as the backbone. The skip connections between the encoding and decoding networks combined deep, semantic, coarse‐grained feature maps from the decoder subnetworks with shallow, low‐level, fine‐grained feature maps from the encoder subnetworks. To integrate the different feature scales, we introduced a multi‐scale attention mechanism, including a spatial attention mechanism and a channel attention mechanism to replace the usual skip connections. The main architecture of the CNN network is shown in Figure 2 and explained as follows: first, feature extraction and context capture of the CT image are performed by the encoder network. These features from different layers containing different spatial and channel information are used as outputs of the attention module (Convolutional Block Attention Module, CBAM).^25^ Second, the attention mappings are concatenated to the corresponding decoding networks, which achieve to predict and locate the labels accurately using different dimensional features. Third, residual connections were introduced in the encoder and decoder networks to address the gradient degradation problem.^26^

### 2.6 Training details

The network architecture was implemented on Keras package with an NVIDIA Tesla P100 graphics card, and trained using a momentum gradient optimizer. The hyperparameters of the Adam optimizer was set to β1 = 0.9, β2 = 0.999. The initial learning rate was 0.01, and weight decay factor was 0.8. 1‐Dice loss was adopted in this study.

### 2.7 Segmentation evaluation metrics

Five segmentation evaluation metrics, including Dice score, Jaccard, Precision, Recall, and Hausdorff distance, were used to evaluate the segmentation accuracy. The evaluation metric formula are as follows:

(1) $$D\mathrm{ice}\left(A,B\right)=\frac{2\left|A\cap B\right|}{\left|A\right|+\left|B\right|}$$

(2) $$Jaccard\left(A,B\right)=\frac{\left|A\cap B\right|}{\left|A\cup B\right|}$$

(3) $$\mathrm{Pr}ecision=\frac{TP}{TP+FP}$$

(4) $$Recall=\frac{TP}{TP+FN}$$

(5) H(A,B)=max(h(A,B),h(B,A))

(6) $$h\left(A,B\right)=\max_{a\in A}(\min_{b\in B}||a-b||)$$

Among them, A represents the prediction contour, and B represents the truth label. TP, FN, and FP are true positives, false negatives, and false positives, respectively.

### 2.8 Fine‐tuning process

We used an uncertainty parameter to select the lowest confidence images in the current network model. The uncertainty calculation formula is as follows:

(7) $$u\;\left(i,j\right)=\left\{\begin{array}{c} p\left(i,j\right),\;0\leq p\left(i,j\right)\leq 0.5\;\\ 1-p\left(i,j\right),\;0.5<p\left(i,j\right)<1\\ 0,\;p\left(i,j\right)=1 \end{array}\right.\;$$

where p(i,j) is the probability map generated by CNN model, while u(i,j) represents the uncertainty of the pixel (i,j). The uncertainty map is quite closely related to the performance of auto‐delineation.^27^

Since the higher uncertainty area located close to the boundary of contours, the second‐order Laplacian operator was used to detect edges. The equations are shown as follow:

(8) $$\begin{array}{ccc} \nabla^{2}f & = & \frac{\partial^{2}f}{\partial x^{2}}+\frac{\partial^{2}f}{\partial y^{2}}=[f\left(x+1,y\right)+f\left(x-1,y\right)\\ & & +\;f(x,y+1)+f(x,y-1)]-4f(x,y) \end{array}$$

(9) $$G\left(i,j\right)=\nabla^{2}f\left\{\left.u(i,j)\right\}\right.$$

**FIGURE 3:**
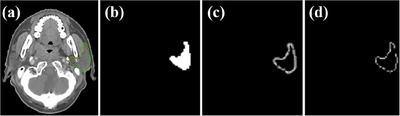
(a) The left parotid contour on CT image, (b) the predicted probability map p(i,j), (c) uncertainty map u(i,j) and (d) G(i,j): the edge area of uncertainty map detected by using second‐order Laplacian operator.

G(i,j) represents the edges of the uncertainty map u(i,j). p(i,j) and G(i,j) are shown in Figure 3. The second order differential emphasizes the mutation of the gray level of the image, and the area where the gray level changes slowly is not concerned. In this way, it can effectively reduce noise interference and have a stronger edge locating ability.

To select candidate samples of worthiness, we calculated the average uncertainty value of each G(i,j), and Formula 10 was as follows:

(10) $$U_{n}=\frac{1}{m}\sum_{1}^{m}G{\left(i,j\right)}_{n}$$

where $U_{n}$ represents the average uncertainty value of the n‐th image, and m represents the pixels number of the n‐th image. The more accurate the segmentation result, the lower the $U_{n}$ value. Thus, we need to choose the sample with the most uncertainty in the current network, and this will make the CNN model learn more valuable and diverse features. After calculating the average uncertainty value of each image, we select the top 30% of $U_{n}$ and add them to the training set for fine‐tuning. The overall iterations were two so that about 50% of the public data set was added to the training set.

### 2.9 Statistical analysis

The paired t‐tests were performed to compare Dice values between HN‐Net and AI software. The significance was determined at *p* < 0.05. All analyses were performed by using SPSS version 23.0 software.

## 3 RESULTS

The segmentation accuracies of four different AI software (designated as A, B, C, and D) were compared with the manual delineations performed by two senior radiation oncologists in the local Hubei Cancer Hospital dataset. A total of seven OARs, including brain stem, esophagus, mandible, left parotid gland, optic chiasm, left optic nerve and pharyngeal constrictor, were delineated. The Dice and HD values of the auto‐segmentations are shown in Tables 1 and 2. The AI software showed a maximum difference of 0.13 and 0.7 mm in average Dice and HD values, respectively. The results showed that there is a large inconsistency between the commercial software. With regard to the individual OAR, the average Dice and HD values ranged from 0.40 ± 0.14 to 0.82 ± 0.04, and (2.7 ± 0.4) mm to (7.2 ± 2.5) mm, respectively. The average Dice value of brain stem, esophagus and mandible are generally higher and more accurate, followed by parotid gland, and pharyngeal constrictor. Small organs such as optic nerve and optic chiasm, there are large differences. Table 2 displayed that the average HD value of brain stem and optic nerve was the smallest, and those of mandible and parotid gland were the largest. The results demonstrated that software A had the highest average Dice value and the lowest average HD value of all included OARs. Even so, the Dice value of optic chiasm in software A was 0.52 ± 0.18 and the HD value of mandible is 7.2 ± 3.0 mm, which is still difficult to be used in our clinical practice without modification.

The performance of the proposed HN‐Net was compared with AI software and other state‐of‐the‐art methods in Tables 3 and 4, based on the Hubei Cancer Hospital data set. Five‐fold cross‐validation was performed to display the final results. The HN‐Net model achieved the best result for the majority of OARs, with an average Dice value of 0.14 higher than the AI software. In particular, the improvement of the optic chiasm and the optic nerve are about 16% to 25% of the Dice value higher than the AI software (*p* < 0.001), suggesting that the re‐training using the local dataset is essential to improve small anatomies with large variations and unclear boundaries. HN‐Net also outperformed other popular networks such as U‐Net,^28^ U‐Net++,^29^ and multi‐scale U‐Net^30^ (HN‐Net: 0.79, U‐Net: 0.78, U‐Net++: 0.78, U‐Net‐Multi‐scale: 0.77). Five evaluation indicators, Dice, Jaccard, Precision, Recall and HD values were performed to test the segmentation ability of HN‐Net, which are shown in Table 4. The average Dice, Jaccard, Precision, Recall and HD values were 0.79, 0.69, 0.88, 0.77 and 2.6 mm, respectively.

**FIGURE 4:**
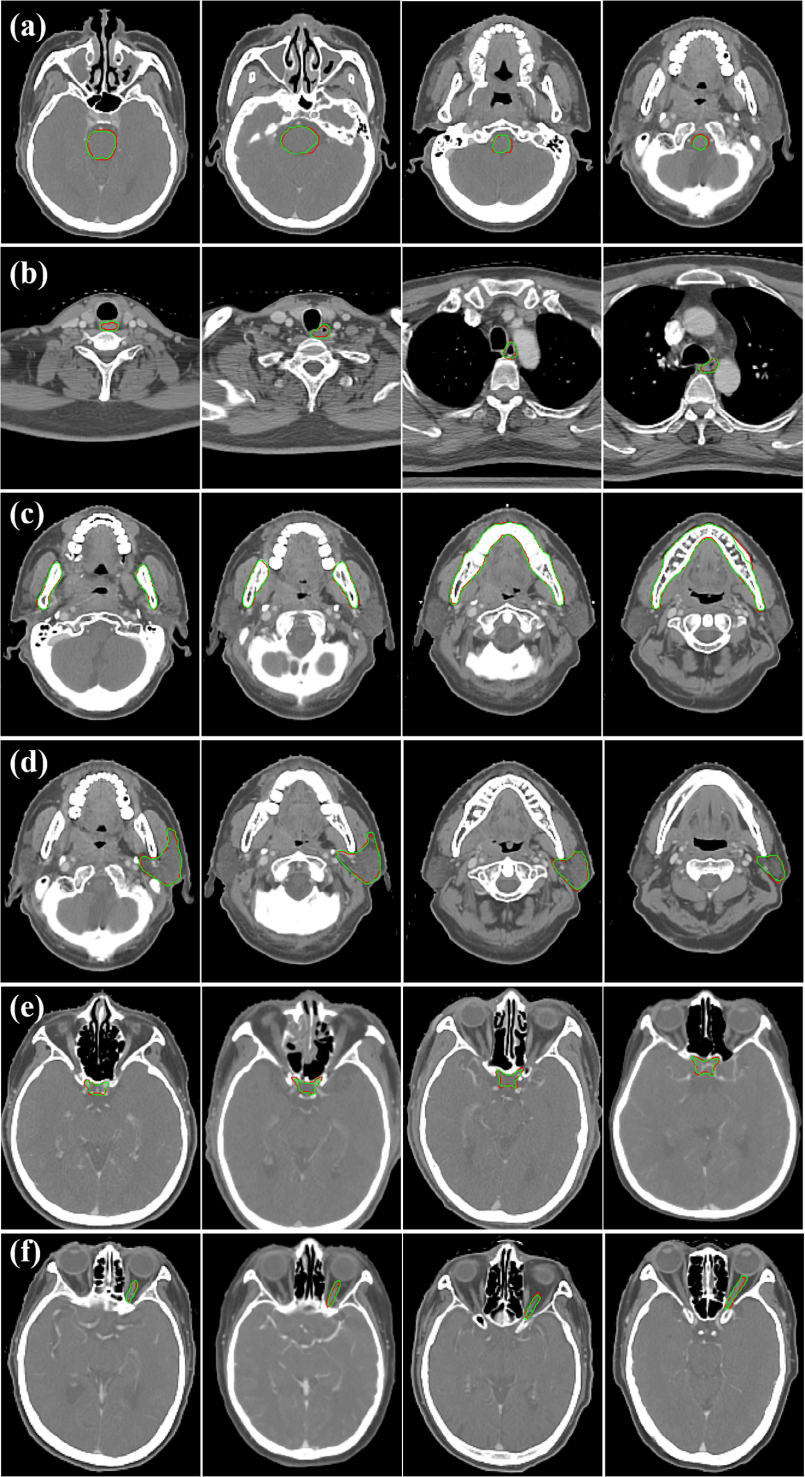
Segmentation results for (a) brain stem, (b) esophagus, (c) mandible, (d) parotid, (e) optic chiasm and (f) left optic nerve with HN‐Net + fine‐tuning model. Red lines denote the manual ground truth contours, and green lines represent auto‐segmentation results.

**FIGURE 5:**
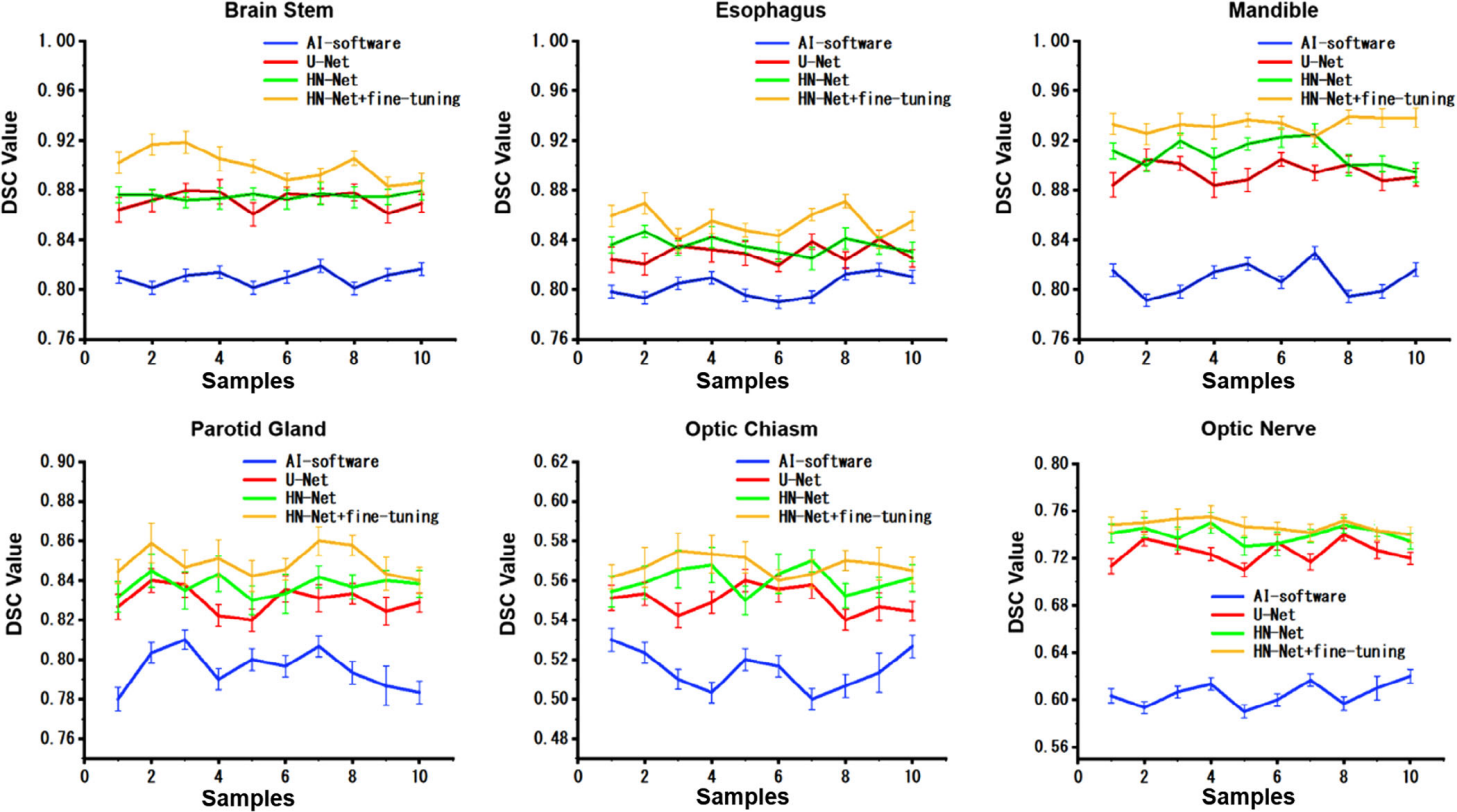
The average Dice value of segmentations from AI software, U‐Net, HN‐Net, and HN‐Net+fine‐tuning model against ten testing samples of Hubei Cancer Hospital. The horizontal axis represents the ten samples from the local testing dataset.

The auto‐segmentation Dice results of HN‐Net fine‐tuning workflow were shown in Table 5. The testing dataset was combination of Québec Public Dataset and 10 samples of Hubei Cancer Hospital. It can be seen that the CNN model plus fine‐tuning has achieved a better segmentation accuracy for both U‐Net and HN‐Net. Figure 4 shows the 2D visualizations of the HN‐Net fine‐tuning model on testing CT images. Red lines are manual contours, and green lines represent auto‐delineations. From the figures, the predicted contours are overall close to the ground truth, especially smaller organs can also be effectively detected and segmented. We have also plotted the Dice score against the ten testing samples from local clinic in Figure 5 to intuitively demonstrate the performance gain. The blue, red, green, and yellow lines represent the AI software, U‐Net, HN‐Net, and HN‐Net plus fine‐tuning, respectively. The results indicate that the fine‐tuning model algorithm can significantly improve the segmentation accuracy and robustness.

## 4 DISCUSSION

Accurate delineation is the basis for precise radiotherapy, which aims to deliver high radiation dose to the tumor target while protecting the healthy tissues. Developing accurate automated segmentation methods is essential for treatment planning and image‐guided radiotherapy. There is no consensus on which auto‐segmentation model works best for clinical applications due to the different structures of OARs and the wide variety of segmentation algorithms. There are several end‐to‐end commercial AI software for automatic segmentation, which saved a lot of contouring time. However, our results showed that there were obvious inconsistencies between different AI software for the same testing dataset, which affected the clinical use. The AI software showed a maximum difference of 0.13 and 0.7 mm in average Dice and HD values, respectively, for selected seven OARs. The average absolute Dice value for brain stem, esophagus, mandible, left parotid gland, optic chiasm, left optic nerve and pharyngeal constrictor were 0.82, 0.70, 0.81, 0.71, 0.40, 0.49, and 0.64, respectively, which are lower than the reported results.^2^, ^17^, ^19^, ^31^ The reason may be due to the use of different CNN structures and different datasets during training. Therefore, it is necessary to re‐train the commercial CNN models based on the local dataset.

Gold standard labels during training are essential for CNN's performance. Although most AI software used the U‐Net as a backbone, some segmentations on local testing datasets were still unacceptable. In this work, we also used the U‐Net, U‐Net++, multi‐scale U‐Net, and HN‐Net to train and test on our local dataset. The dice value results were much higher than those of the AI software, suggesting that there are significant inconsistencies in the training data between the local training and the AI software. The main reason may be that the local gold standard labels were delineated by oncologists according to the same guiding criteria. This will reduce the confusing features learned by the CNN model. Given the same training data, the proposed HN‐Net model outperformed other state‐of‐the‐art methods. The multi‐scale attention module and the residual structure could slightly improve the segmentation accuracy of the model.

The size of the training data is also important for the performance of CNN models. More well‐labeled training data helps improve the performance of the model by covering wider data distribution. After accumulating more new data and using them for re‐training, the performance of the model may degrade as the previous leaned knowledge is forgotten. Therefore, proper fine‐tuning is required. To increase the sample size and improve the robustness of our model, we didn't add the samples randomly. An uncertainty candidate selection method was used to select the valuable and diverse samples for further fine‐tuning. The results showed that the local retraining and fine‐tuning method could further improve the network's generalization ability and segmentation accuracy. Zhu pointed out that a larger training dataset leads to better accuracy on average, except for some structures.^19^ Our proposed HN‐Net and fine‐tuning workflow achieved better accuracy for all selected OARs. This method is similar to the continual leaning with less labeling effort.^32^

In contrast to Attention‐Unet,^33^ HN‐Net employs a distinctive attention strategy by focusing attention primarily on the highest scale of both the encoder and the decoder. This design is computationally more efficient than the incremental layer‐wise approach and places more emphasis on features with global information (global receptive field). In addition, we introduce the CBAM^25^ attention model, which differs from the attention gates in Attention‐Unet. CBAM is able to infer from two different dimensions (spatial and channel dimensions) for the same input, leading to improved feature aggregation and semantic reasoning. For more details on HN‐Net and the attention model we adopted, please refer to our previous work.^34^, ^35^

In the fine‐tuning process, we selected the top 30% of the uncertainty images to retrain the model. When we selected the top 60% or 90% of uncertainty images, it is found that the DSC values showed a minor performance improvement, while the training time escalated significantly. This suggests that the additional computational cost does not translate proportionally into performance gains. Considering this phenomenon holistically, we believe that limiting the selection to the top 30% of uncertain samples strikes the optimal balance between computational efficiency and performance improvement. In addition, the fine‐tuning process is repeated twice. In contrast to selecting 50% of the public samples in a single instance, although both scenarios involved fine‐tuning the neural network using the same size of samples, our staged training strategy allows the neural network to gradually adapt to the data. This strategy is similar to the progressive adaptation of neural networks used in progressive refinement and deep supervision strategies.^36^

In deep learning, fine‐tuning is a usual approach to transfer learning in which the weights of a pretrained model are trained on new data. Models that are pre‐trained on large dataset are usually fine‐tuned by reusing the model's parameters as a starting point and adding a task‐specific layer trained from scratch.^37^ For example, Ma et al. fine‐tuned segment‐anything‐model (SAM) for medical image segmentation through freezing the image encoder and prompt encoder and only fine‐tune the mask decoder.^38^ In recent publications, we and co‐authors used contrastive learning strategy to pre‐train encoder weights on public datasets and transfer them to local tasks for fine‐tuning.^35^ We lack specific network architectures and training information for these commercial AI software, and we also don't know whether they initially employed fine‐tuning for transfer learning. Therefore, it's challenging to make direct comparisons by applying our fine‐tuning methods to the commercial AI software as well. But from Table 5, the CNN plus fine‐tuning has achieved a better segmentation accuracy for both U‐Net and HN‐Net.

Although this algorithm improves the segmentation accuracy, however, there are several limitations. First, for some small voxel organs such as the optic chiasm, the segmentation accuracy still does not meet the clinical standards. Careful reviewing and modification slice‐by‐slice is required after auto‐segmentation. The extreme data imbalance problem and unclear contour boundary may cause bias. Enlarging local receptive field layers and some prior spatial localization techniques will help to solve this problem.^18^ Second, only CT images were used to train our CNN model. Some low contrast organs, such as the optic chiasm, are difficult to distinguish from adjacent areas. Magnetic resonance imaging (MRI) has better resolution of soft tissue contrast, and could provide more accurate border definitions. MRI and other multi‐modality imaging are expected to improve delineation accuracy in future studies. In addition, the HN‐Net was developed and evaluated primarily using the Dice metric. For some large tumor target segmentations, although the Dice value is high, the Hausdorff distance did not meet the uncertainty criteria. This is because the expansion distance from clinical tumor volume to planning tumor volume in radiotherapy may be 5 mm or less. A new loss or evaluation metric may be required for this specific circumstance.

In summary, the accuracy of AI auto‐segmentation software needs to be verified when it is widely used in the clinic. In addition to carefully checking the contours slice by slice, our study provides a coarse training using small samples of local data and a fine‐tuning workflow using the uncertainty estimation method to select valuable and diverse samples from the public dataset. This method could effectively improve the robustness and delineation accuracy of the CNN model.

## 5 CONCLUSION

The existing inconsistencies of auto‐segmentations between different commercial AI software affect their clinical practice. We developed a novel fine‐tuning workflow based on the multi‐scale lightweight residual CNN with attention module, which could automatically select candidate samples of worthiness from public datasets to improve the accuracy and robustness of the CNN model. The method could be feasibly adopted to the clinic for localized further training by using local datasets and external public datasets.

## AUTHOR CONTRIBUTIONS

LS and WK selected the enrolled patients, performed the code and data analysis. YW, ZB, WW, and XX gave useful discussions. XX and LS designed the study and wrote the manuscript. All authors read and approved the final manuscript.

## CONFLICT OF INTEREST STATEMENT

The authors have no conflicts to disclose.
